# *CD7 *in acute myeloid leukemia: correlation with loss of wild-type *CEBPA*, consequence of epigenetic regulation

**DOI:** 10.1186/1756-8722-3-15

**Published:** 2010-04-14

**Authors:** Sonja Röhrs, Michaela Scherr, Julia Romani, Margarete Zaborski, Hans G Drexler, Hilmar Quentmeier

**Affiliations:** 1 DSMZ-German Collection of Microorganisms and Cell Cultures, Braunschweig, Germany; 2 Department of Hematology, Hemostasis, Oncology and Stem Cell Transplantation, Medical School Hannover, Hannover, Germany

## Abstract

**Background:**

CD7 is a negative prognostic marker in myeloid malignancies. In acute myeloid leukemia (AML), an inverse correlation exists between expression of wild-type *CEBPA *and *CD7*. Aim of this study was to find out whether C/EBPα is a negative regulator of *CD7 *and which other regulatory mechanisms might be involved.

**Results:**

As already described for primary AML cells, the majority of AML cell lines tested were either C/EBPα^+^/CD7^- ^or C/EBPα^-^/CD7^+^. However, the existence of isolated CD7^+ ^cell lines expressing wild-type C/EBPα challenges the notion that C/EBPα acts as a unique repressor of *CD7*. Furthermore, ectopic expression of *CEBPA *did not reduce *CD7 *in CD7^+ ^cells and knock-down of C/EBPα failed to induce *CD7 *in CD7^- ^cells. In contrast, the DNA demethylating agent Aza-2'deoxycytidine triggered *CD7 *expression in CD7^- ^AML and in T-cell lines suggesting epigenetic regulation of *CD7*. Bisulfite sequencing data confirmed that CpGs in the *CD7 *exon1 region are methylated in CD7^- ^cell lines, and unmethylated in CD7^+ ^cell lines.

**Conclusion:**

We confirmed an inverse correlation between the expression of wild-type *CEBPA *and of *CD7 *in AML cells. Our results contradict the hypothesis that C/EBPα acts as repressor for *CD7*, and instead show that epigenetic mechanisms are responsible for *CD7 *regulation, in AML cells as well as in T-cells, the typical CD7 expressing cell type.

## Background

*CCAAT/enhancer binding factor *alpha (*CEBPA*), located on chromosome 19q13.1 encodes a transcription factor that is of importance for granulocytic differentiation [1]. C/EBPα is upregulated during myelomonocytic development and positively affects expression of granulocyte differentiation related genes such as the *G-CSF receptor *(*GCSFR*), *myeloperoxidase *and *neutrophil elastase *(*ELA2*) [2-4]. *CEBPA *mutations are found in 5 - 14% of acute myeloid leukemia (AML) cases [5]. C/EBPα mutant proteins block the effect of wild-type C/EBPα on target genes in a dominant-negative manner [6]. This might be the reason why patients with *CEBPA *mutations and those with a silenced *CEBPA *promoter are found in the same AML subclass according to gene expression profiling [7]. Also expression of the T-cell marker *CD7 *has been associated with *CEBPA *mutations and with *CEBPA *hypermethylation [7,8].

*CD7 *is expressed in 30% of AML cases and CD7 positivity is linked with poor prognosis in myeloid malignancies [9,10]. In healthy individuals, CD7 is expressed on thymocytes, T- and natural killer cells, and progenitors of lymphoid and myeloid cells [10]. Conditional knockout experiments in mice suggest that *Cebpa *is involved in the regulation of *Cd7 *expression: absence of *Cebpa *results in upregulation of *Cd7 *in mouse hematopoetic stem cells, reintroduction of the transcription factor reduces expression of *Cd7 *[7].

We wanted to find out whether CD7 positivity in AML can be explained as consequence of loss or inactivation of wild-type *CEBPA*. Therefore, we externally regulated *CEBPA *expression in AML cell lines and tested whether and how this treatment affected *CD7 *expression.

## Results and discussion

### CEBPA expression and CD7 silencing

Quantitative real-time PCR (qRT-PCR) analysis showed that 42% (23/54) of the AML cell lines tested were *CD7 *positive with expression levels comparable to those of T-cell lines, 28% (15/54) of cell lines were weakly positive and 30% (16/54) were *CD7 *negative.

**Figure 1:**
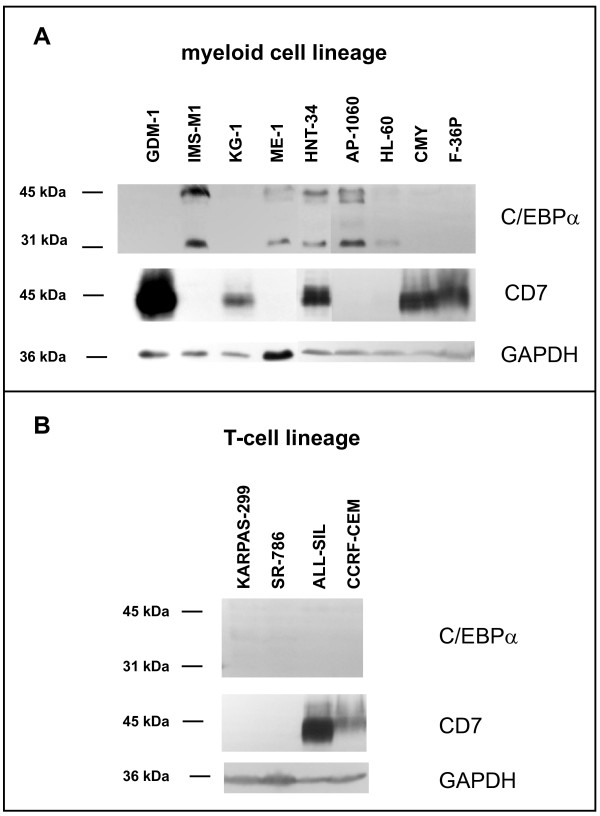
**C/EBPα and CD7 protein expression**. Western blot analyses were performed to detect C/EBPα and CD7 in (A) AML cell lines and in (B) T-cell lines. GAPDH was used as protein loading control. Note that the AML cell line HNT-34 expresses C/EBPα and CD7 and that T-cell lines can be C/EBPα^-^/CD7^-^.

On the first view, Western blot analyses confirmed that C/EBPα might be a negative regulator for *CD7 *expression in AML cell lines: most cell lines showed mutually exclusive expression of these proteins, being either C/EBPα^+^/CD7^- ^or C/EBPα^-^/CD7^+ ^(Fig. 1A, Table 1). However, there was one noticeable exception: cell line HNT-34 expressed both proteins, C/EBPα and CD7, challenging the automatic linkage of C/EBPα expression to *CD7 *repression (Fig. 1A, Table 1).

**Table 1:** C/EBPα and CD7 expression in AML cell lines

C/EBPα	CD7 neg	CD7 low	CD7 high
neg	0	1	9
low	1	1	1
high	10	1	1

Inverse correlation between C/EBPα and CD7 protein expression in 25 AML cell lines as assessed by Western blot analysis. Cell lines classified as "low" showed faint signals, "high" denotes all positive signals in Fig. 1. GAPDH was used as protein loading control. Note that one cell line (HNT-34) expressed C/EBPα (high) and CD7 (high).

Furthermore, it remained open as to how the transcription factor C/EBPα could inhibit expression of *CD7*. The search for transcription factor binding sites using bioinformatic databases (TFSEARCH and TESS) did not reveal a potential C/EBPα binding site in the *CD7 *promoter region (-713 to +624). A report describing that *c-Myc *expression was negatively regulated by C/EBPα via an E2F binding site [11] indicates the possibility of a C/EBPα-mediated transcriptional gene regulation by protein/protein interaction. *CD7 *exon 1 also contains an E2F binding site according to TFSEARCH results. To test whether C/EBPα acts as direct or indirect transcriptional repressor for *CD7 *- by protein/DNA or by protein/protein interaction - we checked our cell lines for any C/EBPα/*CD7*mRNA correlation. Analysis of C/EBPα protein and *CD7 *mRNA expression showed an even weaker correlation than the protein/protein analysis: 3/25 cell lines (HNT-34, IMS-M1, ME-1) were C/EBPα-positive and still showed high expression levels of *CD7 *mRNA (Table 2).

**Table 2:** C/EBPα and CD7 expression in individual AML cell lines

Cell lines	*CEBPA *mRNA	C/EBPα protein	*CD7 *mRNA	CD7 protein
AP-1060	+	+	-	-
CMK	(+)	-	+	+
CMY	-	-	+	+
F-36P	-	-	+	+
GDM-1	(+)	-	+	+
HL-60	+	+	-	-
**HNT-34**	+	+	+	+
HT-93A	+	+	-	-
**IMS-M1**	+	+	+	-
KG-1	(+)	-	+	+
M-07e	-	-	+	+
**ME-1**	+	+	+	-
MEGAL	(+)	-	+	+
MOLM-16	-	-	+	+
MONO-MAC-6	+	+	(+)	-
MUTZ-8	+	+	(+)	(+)
NB-4	+	+	-	-
OCI-AML2	+	+	-	-
OCI-AML5	+	+	-	-
OCI-M1	(+)	(+)	-	(+)
OCI-M2	(+)	-	-	(+)
SET-2	(+)	(+)	+	+
SIG-M5	+	+	-	-
SKNO-1	(+)	(+)	(+)	-
TF-1	-	-	+	+

C/EBPα and CD7 Western blot analysis: + positive, (+) weakly positive, - negative. GAPDH was used as protein loading control. *CEBPA *qRT-PCR: +: 2^-ΔΔct ≥ 5.0; (+) 2^-ΔΔct ≥ 0.2, < 5.0; -: 2^-ΔΔct < 0.2. The *CEBPA*-low cell line SET-2 was used as calibrator cell line. *CD7 *qRT-PCR: +: 2^-ΔΔct > 2.5; (+) 2^-ΔΔct ≥ 1; -: 2^-ΔΔct < 1. The *CD7*-low cell line MUTZ-8 was used as calibrator cell line. Note that cell lines HNT-34, IMS-M1 and ME-1 (bold) express C/EBPα and *CD7 *mRNA.

We sequenced the *CEBPA *gene to find out whether *CD7 *expression in these three cell lines might result from inactivating *CEBPA *mutations. Two of the three C/EBPα^+^/*CD7*^+ ^cell lines (HNT-34, IMS-M1) carried and expressed an in-frame *CEBPA *mutation resulting in four (instead of three) histidine-proline repeats in the transactivation domain 2 of the protein. However, this mutation is considered insignificant for leukemogenesis as it was detected in 39% of healthy volunteers and in 20% of AML patients who remained positive after complete remission [8]. Accordingly, 7/25 (28%) cell lines in our study carried this length polymorphism. Sequencing revealed that none of the three C/EBPα positive and *CD7 *mRNA positive cell lines showed an inactivating *CEBPA *aberration. Furthermore, cell line ME-1 did not carry any mutation at all, showing that the *CD7 *gene could be transcribed despite expression of wild-type C/EBPα.

### No direct influence of C/EBPα on expression of CD7

We had started this project to find out whether CD7 positivity in AML might be due to loss or inactivation of wild-type *CEBPA*. In line with the idea of a repressor function for C/EBPα was the observation that most cell lines showed an inverse correlation between C/EBPα and CD7 expression (Table 2). However, 3/25 cell lines (HNT-34, IMS-M1, ME-1) were C/EBPα^+ ^and still expressed *CD7 *mRNA. C/EBPα/CD7 double positivity does not necessarily contradict a repressor function of C/EBPα. Cell lines HNT-34, IMS-M1 and ME-1 might carry additional genetic or epigenetic alterations not allowing a "normal" repressor function of C/EBPα in these cell lines.

**Figure 2:**
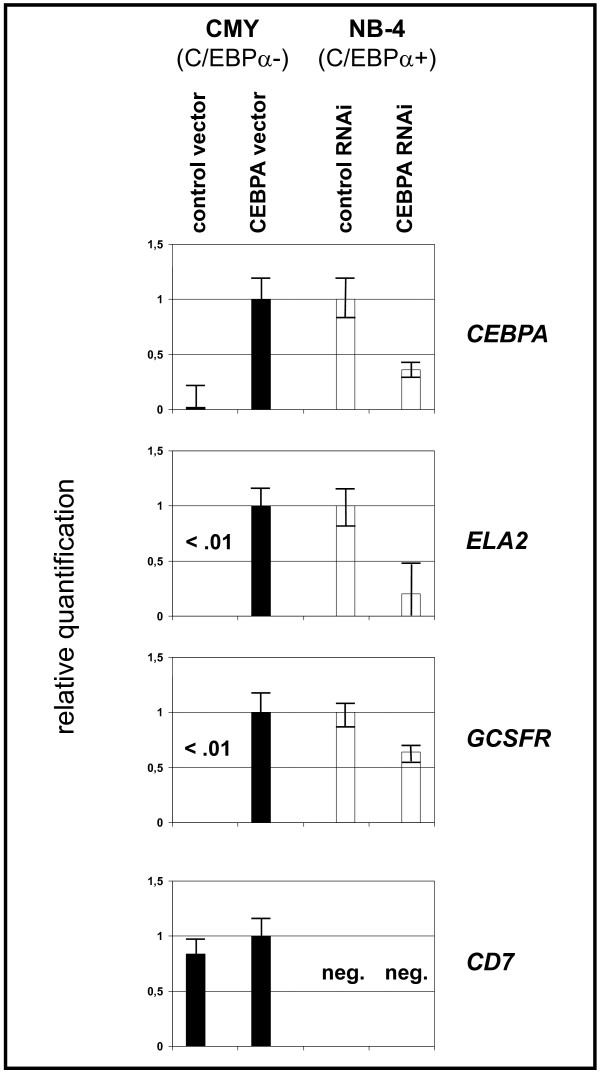
**C/EBPα does not affect *CD7 *mRNA expression**. *CEBPA *was ectopically expressed in the *CEBPA*-negative cell line CMY and repressed in the *CEBPA*-positive cell line NB-4. Expression levels were assessed at day 4 (*CEBPA *RNAi) and day 13 (ectopic expression of *CEBPA*) by qRT-PCR. Gene names at the right-hand side of the figure apply for all four columns. Expression of the C/EBPα targets *ELA2 *and *GCSFR *depends on *CEBPA *expression, *CD7 *mRNA levels are unaffected and remain positive in cell line CMY, negative (neg.) in cell line NB-4.

To experimentally test whether C/EBPα has a direct inhibitory effect on *CD7 *expression, we first ectopically expressed *CEBPA *in the C/EBPα^-^/CD7^+ ^cell line CMY and then knocked down C/EBPα in the C/EBPα^+^/CD7^- ^cell line NB-4 (Table 2). In both cell lines, expression of the transcriptional C/EBPα targets *GCSFR *and *ELA2 *was positively correlated with *CEBPA *expression levels (Fig. 2). In contrast, *CD7 *mRNA levels were neither positively nor negatively affected by C/EBPα (Fig. 2). These results contradict the hypothesis that C/EBPα acts as *CD7 *suppressor.

### Epigenetic regulation of CD7

**Figure 3:**
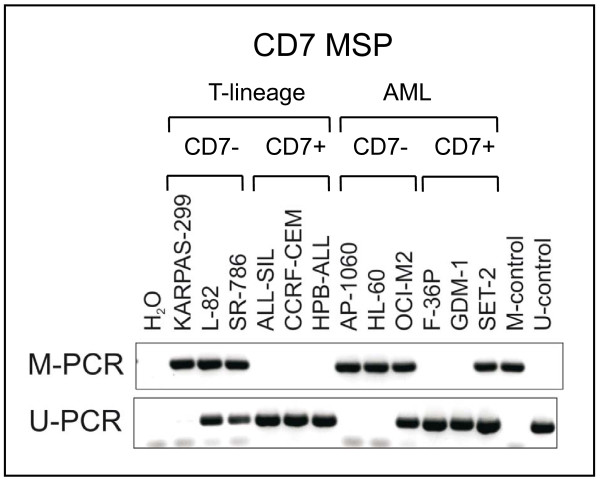
**Epigenetic regulation of *CD7***. *CD7 *promoter methylation was analyzed by MSP after bisulfite conversion of the DNA. Agarose gels of *CD7 *M- and U-PCR products from T- and AML cell lines are shown as representative results.

**Figure 4:**
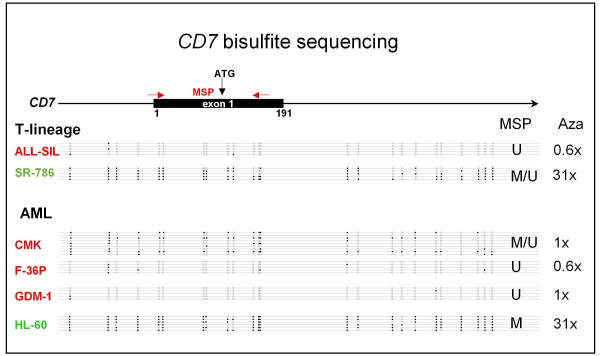
**Bisulfite sequencing of *CD7 *exon 1 region**. CpGs are represented as open dots (U = unmethylated) or filled dots (M = methylated). Name in red: CD7^+ ^cell line, name in green: CD7^- ^cell line. Numbers on the right hand side show the effect of Aza (3 d, 5 μM) on *CD7 *mRNA levels, as assessed by qRT-PCR. Note that Aza induces *CD7 *expression in methylated cell lines (SR-786 and HL-60) only.

Study of T-cell lines confirmed that *CD7 *repression can occur in the absence of C/EBPα: T-cell lines are C/EBPα-negative, but not all T-cell lines express CD7 (Fig. 1B). The *CD7 *promoter region does not match the criteria of a standard CpG island with a GC content > 50% and an observed CpG/expected CpG ratio > 0.6 [12]. However, according to the criteria of Weber *et al*. [13] the *CD7 *exon 1 region contains a subthreshold CpG island (intermediate CpG promoter) with moderate CpG richness (observed CpG/expected CpG ratio > 0.2) and high GC content (> 60%) suggesting that the gene might be epigenetically regulated. Methylation-specific PCR (MSP) and sequencing of bisulfite-converted DNA revealed that this site was methylated in CD7-negative T-cell lines, while CD7-positive T-cell lines were not methylated around the transcriptional start site (Fig. 3, 4). These data support a recent study linking *CD7 *expression to chromatin modifications in CML [14]. Also in AML, unmethylated cell lines (unmethylated signal U only) expressed *CD7 *(2/2), while methylated cell lines (methylated signal M only) were *CD7*-negative (8/9) or weakly positive (1/9) (Table 3). Furthermore, a DNA demethylating agent induced *CD7 *expression in *CD7*-methylated cell lines, independent of histological origin: the T-anaplastic large cell lymphoma-derived cell line SR-786 and the AML cell line HL-60 each showed a ca. 30-fold increase of *CD7 *expression after treatment with 5-Aza-2'-deoxycytidine (Aza), while unmethylated cell lines (ALL-SIL, F-36P, GDM-1) were unaffected (Fig. 4). These results suggest that epigenetic mechanisms play a role in the regulation of *CD7*, both in T-cell lines and in AML cell lines: (i) we found a negative correlation between *CD7 *promoter methylation and gene expression, and (ii) observed that a demethylating agent induced *CD7 *expression in silenced cell lines.

**Table 3:** *CD7 *promoter methylation and *CD7 *mRNA expression in AML cell lines

*CD7 *MSP	*CD7 *mRNA negative	*CD7 *mRNA low	*CD7 *mRNA high
U	0	0	2
U/M	1	2	11
M	8	1	0

*CD7 *methylation was assessed by MSP. U: products with primers recognizing unmethylated *CD7 *only; U/M: U and M (methylated) products; M: M products only. *CD7 *mRNA expression was assessed by qRT-PCR and evaluated as described in Table 2. Note that 2/2 unmethylated (U only) cell lines are *CD7*-positive and that 8/9 methylated (M only) cell lines are *CD7*-negative. Methylation-specific (M) and unmethylation-specific (U) PCR were very sensitive, allowing the detection of one methylated cell in 1000 unmethylated cells and *vice versa*. Therefore, a meaningful correlation between *CD7 *methylation and expression was not apparent in U/M cells.

Interestingly four cell lines (IMS-M1, ME-1, MONO-MAC-6, SKNO-1) were *CD7 *mRNA positive but did not express CD7 protein (Table 2). Future studies might show which posttranscriptional mechanisms - including possibly translational inhibition by microRNAs - are responsible for this phenomenon.

## Conclusions

An inverse correlation between *CD7 *methylation and *CD7 *expression was observed in T-cell lines as well as in AML cell lines suggesting that in both lineages epigenetic mechanisms underlie *CD7 *regulation. Two observations imply that other factors are also important for *CD7 *expression: (i) the stimulating effect of Aza on *CD7 *expression levels varied substantially across different *CD7 *methylated cell lines, and (ii) even cell lines that were clearly responsive to Aza with respect to *CD7 *mRNA induction did not show upregulation of CD7 protein as assessed by Western blot and FACS analysis (data not shown). Although transfection studies did not indicate that C/EBPα acts as *CD7 *repressor, the inverse correlation between *CEBPA *and *CD7 *expression reported for primary AML cases was confirmed for most AML cell lines. Thus, future studies should address whether C/EBPα is a second factor responsible for repression of *CD7 *besides promoter methylation.

## Methods

### Human cell lines

The continuous cell lines were either taken from the stock of the cell bank (DSMZ - German Collection of Microorganisms and Cell Cultures) or were generously provided by the original investigators. Detailed references and cultivation protocols have been described previously [15].

### Methylation-specific polymerase chain reaction (MSP)

Bisulfite conversion of DNA was performed as described by the supplier (EpitTect Bisulfite Kit, Qiagen, Hilden, Germany). For detecting *CD7 *promoter methylation, we performed nested PCR with first round primers (*CD7 *BSP fwd 5'-TTT TGT GGA GAT GTA GGG GTA-3', *CD7 *BSP rev 5'-CAC CAT CAA TCT AAC CAA AAA AAC-3') amplifying converted DNA independently of the methylation status (bisulfite-specific PCR, BSP), while second round primers (*CD7 *M fwd 5'-TTT TTG AGT TTT GAG CGT TTG C-3', *CD7 *M rev 5'-AAA CAA ACC GCG AAC CAA CG-3', *CD7 *U fwd 5'-GTT TTT TTT GAG TTT TGA GTG TTT GT-3', *CD7 *U rev 5'-CCA AAC AAA CCA CAA ACC AAC A-3') for M- and U-PCR specifically recognized the methylated or unmethylated versions of the promoter. PCR products of the initial BSP were diluted 1:100 for subsequent M- and U-PCR. Annealing temperatures were 53°C for BSP with 35 cycles and 63°C for M- and U-PCR with 30 cycles each. Epitect PCR Control DNA (Qiagen) was used as control for methylated and unmethylated templates.

### Bisulfite sequencing

To confirm the methylation status of the *CD7 *promoter, DNA of cell lines was bisulfite converted according to the manufacturer's instructions (Qiagen). Subsequently, amplification of the *CD7 *exon 1 region (760 bp) was performed using primers *CD7 *BSP fwd and *CD7 *BSP rev, specifically binding bisulfite converted DNA (for primer sequence and PCR conditions see MSP section). Resulting *CD7 *fragments were purified, cloned into pGEM-TEasy vector (Promega, Madison, WI, USA) and sequenced. Sequences were evaluated using BiQ Analyzer http://biq-analyzer.bioinf.mpi-sb.mpg.de and had to conform to at least 90% bisulfite conversion rate [16]. In addition, identical clones were excluded from the analysis.

### Gene expression analyses

Quantitative PCR was performed on a 7500 Applied Biosystems (Darmstadt, Germany) real-time PCR system using the manufacturer's protocol. RNA was prepared using the RNeasy Mini kit (Qiagen). For mRNA quantification, reverse transcription was performed using the SuperScript II reverse transcriptase kit (Invitrogen, Karlsruhe, Germany). TaqMan probes (Applied Biosystems) were used to quantify human *CEBPA *(Hs 00269972s1), *ELA2 *(Hs 00357734 m1) and *GCSFR *(Hs 01114427 m1) expression levels with *TBP *as endogenous control. Expression of *CD7 *was assessed using the SYBR GREEN PCR Master Mix (Applied Biosystems) with *GAPDH *as internal control. *CD7 *forward: 5'-GTG CTG GCG AGG ACA CAG-3'; *CD7 *reverse: 5'-TCG TAC ACC ACA CAT GCC G-3'. *GAPDH *forward: 5'-TGG GTG TGA ACC ATG AGA AG-3'; *GAPDH *reverse: 5'-TCC ACG ATA CCA AAG TTG TCA-3'. Relative expression levels were calculated using the ΔΔCt-method.

### Treatment with DNA demethylating agent Aza

5-Aza-2'-deoxycytidine (Aza) (Sigma) dissolved in DMSO was used to verify the effect of methylation on expression of *CD7*. Cells were seeded at a cell density of 5 × 10^5 ^cells/ml, Aza was added at a final concentration of 5 μM. Control cells were treated with 0.05% DMSO. After 2 d, half of the medium was replenished with medium with/without Aza (5 μM). After 3 d, respectively 4 d, cells were harvested to prepare RNA or protein.

### Western blot analysis

Samples were prepared as described previously [17]. Anti CD7 antiserum was purchased from Santa Cruz (Heidelberg, Germany), anti C/EBPα antiserum was obtained from Cell Signaling/New England Biolabs (Frankfurt, Germany). Specific bands on nitrocellulose membranes were visualized with the biotin/streptavidin-horseradish peroxidase system (Amersham, Freiburg, Germany) in combination with the "Renaissance Western Blot Chemoluminescence Reagent" protocol (DuPont, Bad Homburg, Germany).

### Bioinformatic database search for C/EBPα binding sites

The genomic sequence of the *CD7 *promoter region from -713 to +624 was analyzed with the database search tools TFSEARCH ver.1.3 http://www.cbrc.jp/research/db/TFSEARCH.html and TESS http://www.cbil.upenn.edu/cgi-bin/tess/tess for the existence of potential C/EBPα binding sites (Factor ID in TESS: T00105).

### Plasmid construction

For generating the anti-*CEBPA *shRNA, DNA oligonucleotides corresponding to position 818-836 of the sequence of the human *CEBPA *gene (GenBank accession no. NM_004364.3) were subjected to BLAST homology search, and thereafter chemically synthesized including overhang sequences from a 5'-*Bgl*II and a 3'-*Sal*I restriction site for cloning purposes (BioSpring, Frankfurt, Germany). The numbering of the first nucleotide of the shRNA refers to the ATG start codon. The oligonucleotide sequences were as follows: FP*CEBPA*: 5'-GATCCCCGGCCAAGAAGTCGGTGGACTTCAAGAGAGTCCACCGACTTCTTGGCCTTTTTTGGAAG-3'; RP*CEBPA*: 5'-CGACTTCCAAAAAAGGCCAAGAAGTCGGTGGACTCTCTTGAAGTCCACCGACTTCTTGGCCGGG-3'.

The non-complementary 9-nt loop sequences are underlined, and each sense oligonucleotide harbors a stretch of T as a *Pol*III transcription termination signal. The oligonucleotides were annealed and inserted 3' of the H1-RNA promoter into the *Bgl*II/*Sal*I-digested pBlueScript-derived pH1-plasmid to generate pH1-*CEBPA *as described [18]. The control plasmid pH1-GL4 has been described earlier [18]. Finally, the H1-*CEBPA *expression cassette was excised by digestion with *Sma*I and *Hinc*II and blunt-end ligated into the *Sna*BI site of the pdc-SR lentiviral vector to generate pdcH1-*CEBPA*-SR plasmid. The lentiviral plasmid encodes RFP_EXPRESS _as reporter gene.

### Preparation of recombinant lentiviral supernatants and lentiviral transduction

Preparation of recombinant lentiviral supernatants and transduction were performed as described previously [18]. The titers were averaged and typically ranged between 5-10 × 10^8 ^IU/ml. Concentrated viral supernatants were used for transduction of NB-4 cells in 48-well plates as described [18].

## Competing interests

The authors declare that they have no competing interests.

## Authors' contributions

SR designed parts of the study and performed MSP analysis, sequencing of bisulfite-converted DNA and co-wrote the manuscript, MS performed knock-down and expression experiments, JR performed Western blot analysis, MZ performed quantitative real-time PCR, HGD provided cell lines and critically read the manuscript, HQ designed the study and wrote the manuscript. All authors read and approved the final manuscript.
